# Multi-Layer PVA-PANI Conductive Hydrogel for Symmetrical Supercapacitors: Preparation and Characterization

**DOI:** 10.3390/gels10070458

**Published:** 2024-07-12

**Authors:** Angelica Giovagnoli, Giada D’Altri, Lamyea Yeasmin, Valentina Di Matteo, Stefano Scurti, Maria Francesca Di Filippo, Isacco Gualandi, Maria Cristina Cassani, Daniele Caretti, Silvia Panzavolta, Maria Letizia Focarete, Mariangela Rea, Barbara Ballarin

**Affiliations:** 1 Department of Industrial Chemistry “Toso Montanari”, University of Bologna, Distretto Navile—Via Gobetti 85, 40129 Bologna, Italy; angelica.giovagnoli2@unibo.it (A.G.); giada.daltri2@unibo.it (G.D.); yeasmin.lamyea@unibo.it (L.Y.); valentina.dimatteo5@unibo.it (V.D.M.); stefano.scurti2@unibo.it (S.S.); mariacristina.cassani@unibo.it (M.C.C.); daniele.caretti@unibo.it (D.C.); 2 Applied Science and Technology Department, Politecnico di Torino, Corso Duca Degli Abruzzi, 24, 10129 Torino, Italy; 3 Department of Chemistry “Giacomo Ciamician”, University of Bologna, Via Selmi 2, 40126 Bologna, Italy; maria.difilippo5@unibo.it (M.F.D.F.); silvia.panzavolta@unibo.it (S.P.); marialetizia.focarete@unibo.it (M.L.F.); mariangela.rea2@unibo.it (M.R.); 4 Center for Industrial Research-Advanced Applications, Mechanical Engineering and Materials Technology CIRI MAM University of Bologna, Viale Risorgimento 2, 40136 Bologna, Italy; 5 Center for Industrial Research-Fonti Rinnovabili, Ambiente, Mare e Energia CIRI FRAME University of Bologna, Viale Risorgimento 2, 40136 Bologna, Italy

**Keywords:** hydrogel, conducting polymer, supercapacitor, wearable sensor, flexible materials

## Abstract

This work describes a simple, inexpensive, and robust method to prepare a flexible “all in one” integrated hydrogel supercapacitors (HySCs). Preparing smart hydrogels with high electrical conductivity, ability to stretch significantly, and excellent mechanical properties is the last challenge for tailored wearable devices. In this paper, we employed a physical crosslinking process that involves consecutive freezing and thawing cycles to prepare a polyvinyl alcohol (PVA)-based hydrogel. Exploiting the self-healing properties of these materials, the assembly of the different layers of the HySCs has been performed. The ionic conductivity within the electrolyte layer arises from the inclusion of an H_2_SO_4_ solution in the hydrogel network. Instead, the electronic conductivity is facilitated by the addition of the conductive polymer PANI-PAMPSA into the hydrogel layers. Electrochemical measures have highlighted newsworthy properties related to our HySCs, opening their use in wearable electronic applications.

## 1. Introduction

The constant development of portable technologies is establishing a demand for the integration of smart devices in everyday objects [1,2]. The gap between human life and technology must be compensated with biocompatible and wearable electronics that have the purpose of introducing more accurate and precise procedures [3,4,5,6]. The study of wearable electronics has peaked in recent years, thanks to the advent of commodities, especially in the medical field, as devices that can monitor heart rate, skin temperature, muscle movement, or sense chemical stimuli. The design of these devices requires flexible and deformable power systems that can adapt to the human body in comfortable and biocompatible ways [5,6,7]. Electronic components with high technological maturity struggle to satisfy this demand, as they are based on stiff materials and structures that do not match the characteristics of human tissues. Research efforts will be necessary to answer these challenges. This work proposes a new strategy for the fabrication of flexible and deformable hydrogel-based supercapacitors.

Wearable electronics can be powered by energy-harvesting [8] and energy storage systems [9,10]. Because of discontinuous energy collection, devices with higher energy adsorption must accumulate the energy, which is self-produced or externally supplied, in batteries and/or supercapacitors. Supercapacitors (SCs) are devices that can electrochemically store energy, and efficiently release it with a high density over a relatively short time [11,12]. Depending on their operating principle, supercapacitors are mainly categorized into two types, which are pseudo capacitance and electric double-layer capacitance. Pseudo supercapacitors store energy through electrochemical reactions, while double-layer capacitors (EDLCs) employ predominantly electrostatic charge separation occurring at the interface between the electrode surface and the electrolyte [13]. SCs are extensively utilized in consumer electronics and renewable or industrial energy systems [12]. SCs are promising devices in wearable electronics due to the simplicity, affordability, and safety of their fabrication and operation modes. Traditional structures, composed of metallic electrodes and a liquid electrolyte, experience great difficulties in wearable electronics implementation caused by the physical state of the electrolyte. Firstly, the electrolyte leak represents a concrete risk in terms of toxicity both for humans and the environment, with obstacles in device recycling. Moreover, the presence of an aqueous or organic electrolyte interlayer requires the insertion of a separator in the SC to avoid short-circuit during the real operation that involves different mechanical stimuli. The consequent extra step during fabrication increases the costs and the complexity of the device. Furthermore, mechanical stress negatively affects the adhesion between the electrode material and electrode modifier, both in area and strength, resulting in a decreased performance during operation. The above-mentioned obstacles can be overcome with the implementation of a solid electrolyte wherein the active materials are deposited [14]. Solid flexible compounds, as hydrogels, can replace stiff materials in SC fabrication, resulting in a simple, cost-effective, and reliable solution [10,15].

Hydrogels are three-dimensional crosslinked polymer matrices capable of absorbing aqueous solutions into pores [16,17,18]. Their structure allows them to retain abundantly the electrolyte solution with their hydrophilic nature, while the porous polymer structure creates a preferred path for charges, which provides ionic conductivity like traditional aqueous electrolytes and improves mechanical properties with a minimal cost in terms of electrochemical performances [19]. Indeed, their soft and solid-like structure allows the fabrication of flexible materials, as they can sustain stretching, bending, and twisting deformation while maintaining the original form and retaining electrochemical properties after deformation and recovery cycles. These properties depend on the preparation of the hydrogel, with particular attention to the composition, crystalline phase percentage, type of crosslinking, and water content [18,20]. Because of their crosslinked porous structure, hydrogels are intrinsically amorphous, independently from the composition. They can be categorized as natural or synthetic [20]; hydrogels based on synthetic polymers tend to exhibit tunable mechanical, optical, or electrochemical characteristics with a simpler composition, by tuning a few parameters. Chemically crosslinked hydrogels require specific crosslinker agents, acting as bridges where macromolecules are knotted and bonded [17,18]. The resulting materials are extremely stable and mechanically performant, but lack biocompatibility and recyclability, as the bonds are irreversible and crosslinker agents tend to be toxic [21,22]. Physical crosslinking hydrogels are based on physical interactions between the polymer macromolecules, tend to be more environmentally friendly, and present simplicity in preparation. Among the different materials that are used for hydrogel fabrication, polyvinyl alcohol (PVA) has piqued researchers’ interest because of its chemical stability, nontoxic biocompatible nature, and the possibility to tailor mechanical, electrical, and optical properties by implementing fillers [23,24]. As a macromolecule, the structure is linear and hydrophilic thanks to the presence of hydroxyl groups, allowing the dissolution of the compound in aqueous solutions. Physically crosslinked PVA hydrogels obtained by the establishment of hydrogen bonds between the hydroxyl groups of the chains give rise to the formation of a three-dimensional matrix, with a simple yet effective way of fabricating a material that is valid for various applications, as in medical [23,24,25,26,27] and energy fields [10,28,29,30]. An easily reproducible method to induce physical crosslinking is letting the PVA solution undergo repetitive and alternate freeze–thaw cycles so that the macromolecules align and entangle between the ice crystals formed at freezing temperature [31,32,33]. By alternating freezing cycles, the ice crystals entrapped between the chains melt, and new hydrogen bonds can be formed repeating the process. Key parameters for this method are molecular weight (MW), polymer amount, temperature, duration of each step, and number of cycles. The resulting material presents high stretchability, resistance to deformation, high solution retention, and self-healing properties [32,33]. Recently, our group has studied the role of PVA molecular weight in hydrogel preparation for energy purposes and its influence on electrochemical properties [34].

A fundamental element in SCs is the electrode material. The more consolidated electrodes can hardly be implemented in wearable devices [11,35]. Carbon-based materials and conductive polymers (CPs) have paved new ways to fabricate advanced wearable SCs due to their lightweight, processability, and biocompatibility. In the vast scenario of conductive polymers, the environmental stability, simple synthesis, and interesting optical, electrochemical, and electrical properties of polyaniline (PANI) are characteristics that enhance its usage in newly produced devices [36,37,38]. PANI varies in optical properties during redox processes, making the different states recognizable through color and presenting electrochromic characteristics [39,40]. PANI in its half-oxidized state, emeraldine form [37,41], thanks to its thrilling conductivity, has been implemented in various types of devices and materials as pH [42,43], humidity [44], chemical sensors [45], or electrode material for rechargeable batteries and SCs [46,47]. Due to the high potential, systems that combined the properties of PVA and PANI for developing batteries and SCs are still under study today [32,48,49,50]. In this work, exploiting the self-healing properties of polyvinyl alcohol (PVA)-based hydrogel obtained by a freezing–thawing physical method, a symmetric all-in-one supercapacitor (HySCs) with electrode/electrolyte hydrogel has been prepared. The ionic conductivity within the electrolyte layer arises from the inclusion of an aqueous H_2_SO_4_ solution in the hydrogel network. The addition of polyaniline stabilized with poly(2-acrylamido-2-methyl-1-propanesulfonic acid (PANI_PAMPSA) derives the electronic conductivity of the hydrogel electrode layers [44]. The raw materials used to synthesize the PANI:PAMPSA hydrogel are relatively low-cost and commercially available. This allows for potentially more cost-effective manufacturing of the hydrogel compared to specialized hydrogel formulations. These innovative HySCs have been characterized and tested, presenting newsworthy properties for wearable electronic applications.

## 2. Results and Discussions

### 2.1. Hydrogels Characterizations

The hydrogel electrolytes (Hy_x_) were obtained by a freeze–thaw method which promotes the formation of physical crosslinks via hydrogen bonding interactions [31]. To this purpose, PVA with different molecular weights (31,000–50,000 and 89,000–98,000) have been used for their preparation (H_y1_ and H_y2_ respectively). The hydrogel electrodes (Hy_x-PANI_PAMPSA_) were obtained with the same procedure, but with the addition of the conductive polymer PANI_PAMPSA after the PVA dissolution. Due to the self-healing properties of PVA hydrogels and the integration of different hydrogel layers (i.e., electrode/electrolyte or electrode/electrolyte/electrode configurations), semi-cells and symmetric supercapacitors have been fabricated, as reported in Section 4.

**Figure 1:**
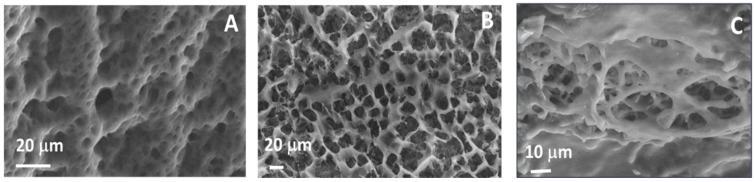
SEM image of (**A**) S2-WL; (**B**) S2-GL; (**C**) enlargement of (**B**) at 500×.

The SEM, TGA, and ATR-FTIR analyses were carried out on the semi-cells (S_x_) with each constituent layer being analyzed individually, subsequently labeled as the white layer (WL = Hy_x_) and the green layer (GL = Hy_x-PANI_PAMPSA_). The internal morphological structure of the single hydrogel layer was investigated by SEM microscopy (Figure 1A).

The images highlighted a notable morphological difference between the electrolyte (S_2_-WL) and the electrode (S_2_-GL) layers, where the electrode layer was characterized by the copresences of two distinct networks [50]. This peculiar morphology was not observed for the WL layers.

The porosity tests conducted on each semi-cell (Figure S1A) showed that the presence of the PANI_PAMPSA in the hydrogel matrix changes its morphology but not the degree of porosity, which is strictly related to the PVA molecular weight. A decreasing porosity can be observed for the hydrogel obtained with lower molecular weight but with a higher amount of PVA (H_y1_), due to a major number of entanglements per unit volume that reduce the pore areas.

Moreover, the thermal stability was investigated by TGA, and the related thermograms reported in Figure S1B, (red line) exhibit three degradative steps in agreement with the studies reported in the literature [51,52]. The first step (50–200 °C) can be attributed to the loss of water residue in the sample, while the second one (200–340 °C) was related to the loss of hydroxyl groups of the polymer matrix. The third degradation step (340–450 °C) can be associated with polymer chain decomposition. Instead, the thermograms of S_x_-GL (in green line) exhibited two main degradative steps: the first in the temperature range up to 200 °C derived from the loss of moisture present in the sample; the second one (400–500 °C) was due to polymeric backbone degradation.

A slight shift in the last step degradation temperature can be observed in the presence of PANI_PAMPSA due to the aromatic structure that influences the hydrogel thermal properties at high temperature [52].

ATR-FTIR spectroscopy was carried out on S_x_ samples previously washed to remove the acidic media and subjected to freeze-drying. By analyzing the compared spectra that are available in the Supplementary Materials, S_x_-WL layers, and the pure PVA show both the characteristic peak at 3400 cm^−1^ corresponding to O-H stretching, the peak at 2950 cm^−1^ typical of C-H stretching, at 1640 cm^−1^ the C-O-H out of plane bending, and lastly the signal at 1090 cm^−1^ corresponding to the C-O stretching [53,54]. Despite the PVA matrix representing the most prominent contributor in the S_x_-GL spectrum, the presence of PANI_PAMPSA in the conductive layers can be confirmed from the peak at 1660 cm^−1^ associated with the C=O stretching of PAMPSA (Figure S2) [50].

Furthermore, the mechanical properties of S_x_ have been investigated with uniaxial tensile tests, and the measured values are reported in Table 1 and Figure S3.

A strict correlation exists between the PVA’s molecular weight and its mechanical properties, where an increase in molecular weight results in a decrease in Young’s modulus and an increase in maximum tensile strain, due to the enhanced likelihood of polymeric chains to form entanglement-type interactions with other chains as the length of the macromolecules increases [55].

The water content (Wc%), the swelling ratio (S_W_%), and the swelling ratio normalized to the polymer fraction (S_PVA_%) were calculated for the Hy_x_ layers as described in Supplementary Materials. The results highlighted good swelling capacity for both PVA hydrogels (Hy_1_ and Hy_2_); the system prepared using PVA with higher molecular weight (Hy_2_) exhibited the most pronounced swelling due to the higher porosity observed. Instead, the symmetric cells (Hy_x_SC) showed a notable correlation with the water content and swelling ratio observed in the constituent hydrogel layers (Table 2).

Moreover, the self-healing property was investigated in our previous work, in agreement with M. Shin et al. [56]. Material can be classified as self-healing if it satisfies three rheological criteria: (i) demonstrates terminal flow pseudoplastic behavior; (ii) possesses a chain flow relaxation time (tf) within a reasonable time scale; and (iii) behaves as a viscous fluid at low frequencies [56]. As described in Supplementary Materials, the slope of the initial dynamic viscosity (*η*′) curve (Figure S4) with an absolute value equal to 0.07 confirms the Newtonian character of the hydrogel at low frequency, typical of self-healing materials (pseudoplastic fluids) [56]. Furthermore, the results of the frequency sweep test (Figure S4B) suggest that the dynamic bond-based gel network forms a pseudo-structure with minimal steric hindrance for the chain flow, owing to the short lifetime of the dynamic bond. Indeed, a tf value of 0.03 s can be calculated for Hyx, given by the reciprocal value of the crossover frequency, confirming that the chain flow relaxation strongly contributes to self-healing on a reasonable time scale (for no self-healing materials tf results impossible to calculate). Moreover, Figure S4C shows an upward trend of tanδ, confirming the hydrogel liquid-like behavior. Finally, the crossover between G′ and G″ occurred with 4.7% of applied shear strain and 8.2 Pa of applied shear stress (Figure S4D). This information was used to perform the three-interval thixotropic test (3ITT) in controlled shear rate (CSR) mode to assess the hydrogel’s mechanical properties (Figure S4E). From the curve, it was possible to calculate the % recovery of the viscosity of the hydrogel after the high shear rate applied, which amounted to 64% after 2 min. The obtained results suggested that Hyx completely fulfilled the three required criteria, from a rheological point of view, to be classified as a self-healing material.

### 2.2. Electrochemical Behavior

To investigate the electrochemical behavior, 4-point conductivity, EIS, and galvanostatic charge/discharge (GCD) measurements were conducted on the semi-cells and then on integrated hydrogel supercapacitors as previously described. All measurements have been performed in triplicate on three different samples at environmental conditions (25 °C).

#### 2.2.1. Semi-Cell and Symmetric Cell

The conductivity of the semi-cells depended on both the specific conductance (κ) for the S_x_-GL, and the ionic conductivity obtained from the EIS measurements on the S_x_-WL samples (Figure S5). The specific conductance comes from electron mobility into the hydrogel containing the conductive polymer. The data reported in Table 3 reveal a higher specific conductance for S_1_ than S_2_.

The ionic conductivity (*σ_c_*) obtained from EIS measurements (Figure S5) highlighted a trend in agreement with the data relating to % porosity previously discussed. The conductivity of the gels is high enough to ensure high currents flowing in the electrolyte. The three-dimensional porous network plays a crucial role in ensuring the flow of ionic charges. Specifically, hydrogel semi-cell S_2_, characterized by higher ionic conductivity, exhibited a porosity greater than S_1_.

**Figure 2:**
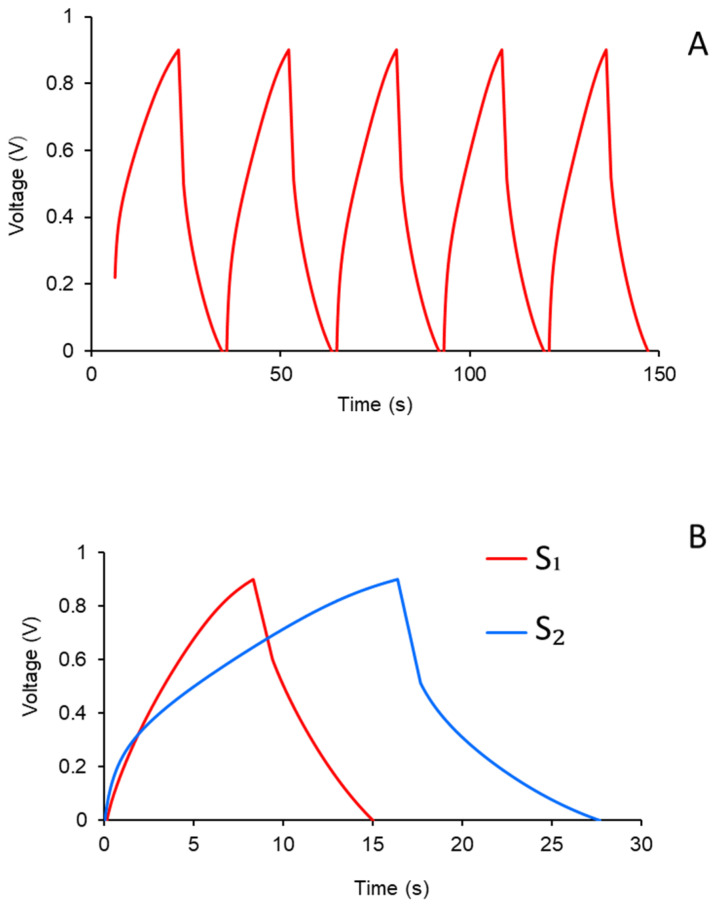
(**A**) GCD curves of S_1_; (**B**) third GCD cycle comparison for S_1_ and S_2_.

The parameter that most effectively assesses the functionality of the SC devices is the specific capacitance (Cp). The semi-cell Cp values have been obtained from galvanostatic charge–discharge (GCD) tests conducted for each semi-cell, at a constant current density of 0.025 mA cm^−2^ within the potential window between 0.00 and 0.90 V vs. SCE (saturated calomel electrode) for five cycles totals. Subsequently, by Equation (2), specific capacitance was calculated as described in Table 3. Figure 2 reported, as an example, the GCD measurements of S_1_ (Figure 2A) and the comparison of the third GCD cycle between S_1_ and S_2_ (Figure 2B).

**Figure 3:**
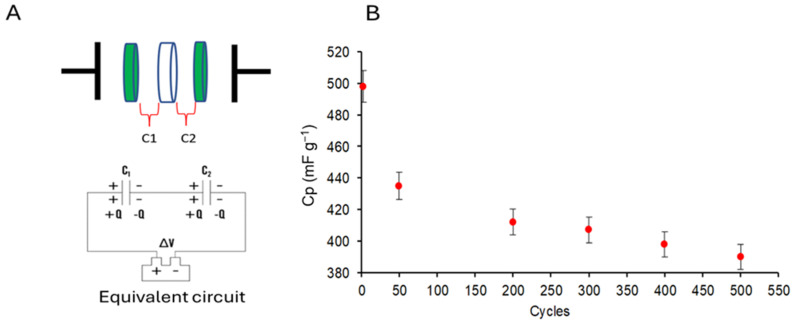
(**A**) Symmetric cell assembly and equivalent circuit; (**B**) specific capacities vs. consecutive cycles (*n* = 3 tested; error bars are reported).

The symmetric cell Hy_x_SC can be considered as an equivalent circuit comprising two capacitances in series (Figure 3A). The equivalent specific capacitance corresponds to the sum of the reciprocals of the capacitances of the system, as described by the following equation, where m is the mass of conductive polymer introduced inside each electrode layer:

(1) $$\frac{1}{C_{eq}}=\frac{1}{C_{1}}+\frac{1}{C_{2}}+\cdots \frac{1}{C_{n}}$$

where *C*_1_ *= C*_2_ *= C*

(2) $$\frac{1}{C_{eq}}=\frac{2C}{C^{2}}=\frac{2}{C\;}$$

(3) $$C_{eq}=\frac{C}{2}=C_{device}$$

(4) $$C_{p}=\frac{C_{device}}{m}$$

Hy_2_SC, obtained from PVA with a molecular weight of 89,000–98,000, resulted in the most efficient charge accumulation at the interface and showed a higher Cp value. In Figure 3B, the trend of specific capacities is calculated on consecutive cycles. After 500 cycles, the specific capacitance reached a value corresponding to 78% of the initial capacitance.

**Figure 4:**
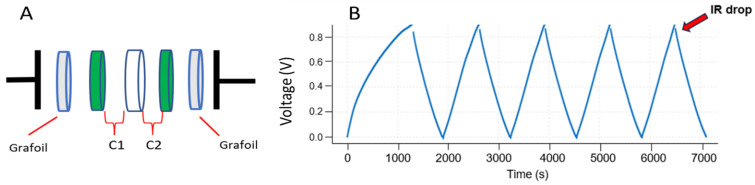
(**A**) Symmetric cell assembly with Grafoil collectors; (**B**) GCD curves.

An alternative to the previous cell has been assembled using two flexible current Grafoil collectors on the external sides of electronic layers. Preliminary tests have been conducted by interposing two 3.0 mm diameter Grafoil disks between the symmetric cell and the Swagelok-type electrodes (Figure 4A). The Grafoil allowed us to increase the Cp values of the symmetric cells (Table 4) and to decrease the IR drop in the GCD curves (Figure 4B).

Moreover, charge and discharge measurements can be conducted by increasing the applied current density to 0.1 and 0.3 mA. As reported in Table 4, the Cp values decreased with increasing current due to the kinetics associated with charge movement. The cell had the same resistances to overcome and was subjected to a higher current density, so it had less time to rebalance, leading to less charge accumulation.

#### 2.2.2. Flexible Supercapacitor Unit Assembly and Application

**Figure 5:**
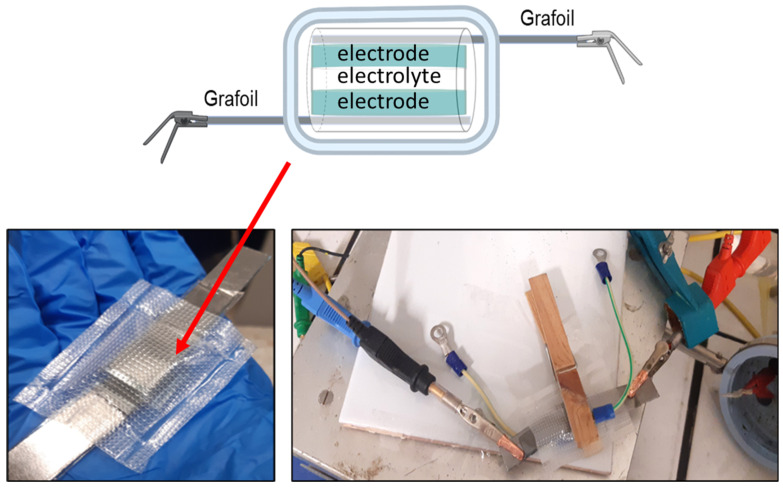
Flexible symmetric integrated hydrogel supercapacitors (HySCs) with corresponding scheme and assembly for GCD measurements.

The flexible supercapacitor (FSC) units were assembled by interposing a square of symmetric cell hydrogel (1 cm^2^) between two strips of Grafoil with a width of 1.0 cm as shown in Figure 5.

The Grafoil acted as a flexible current collector due to its high conductivity. The assembled system was first covered with a transparent inert adhesive tape to enhance adhesion between the layers; finally, it was sealed inside a transparent LDPE (low density-polyethylene) film.

The Cp values obtained for the flexible supercapacitor units were reported in Table 5 and compared with those of PANI-based composite thin films for both flexible and tradition supercapacitor applications reported in the literature [50,57,58,59,60,61,62]. The specific capacitance of Hy_2_SC results are higher than Hy_1_SC and fit well with some values reported in the literature. Although the specific capacitance of our PANI:PAMPSA composite device is approximately four times lower than that of the best traditional PANI-based supercapacitor, it is important to note that the specific capacitance was calculated here considering the total mass of the PANI:PAMPSA composite. Since PAMPSA is non-electroactive, it does not contribute to the overall electrochemical capacity of the composite material, resulting in lower observed specific capacitance values. Moreover, the decrease in specific capacitance observed during cycling is in line with recent literature.

Table 5 highlights how the production of an all-in-one supercapacitor with an electrode–electrolyte composite hydrogel is a promising approach for the fabrication of wearable energy storage devices. The hydrogel structure allows for high ionic conductivity and flexibility, making it well-suited for integration into wearable systems that require conformable, lightweight energy storage. The all-in-one design could simplify manufacturing compared to multi-component supercapacitor assemblies. Importantly, the use of a gel electrolyte in our device represents an advantage over traditional liquid electrolyte supercapacitors, as it significantly reduces the risk of electrolyte leakage—a common issue with liquid-based systems. The integrated hydrogel structure further minimizes the potential for leakage, enhancing the safety and reliability of the wearable energy storage solution.

For a demo usage of Hy_x_SC as flexible supercapacitors, three supercapacitor units have been prepared as previously described and connected to a red light-emitting diode (LED).

**Figure 6:**
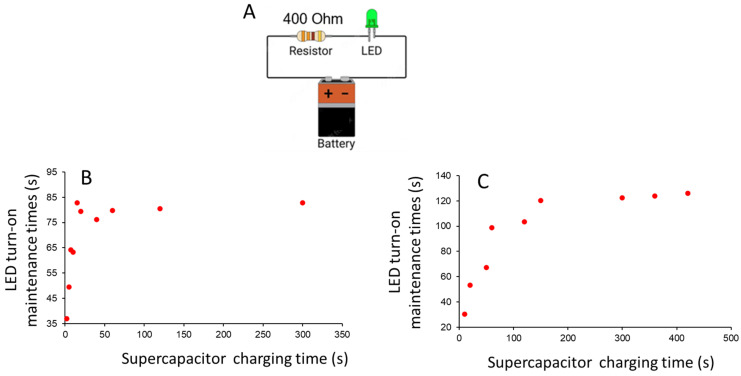
(**A**) Equivalent circuit for LED power on; (**B**) LED turn-on maintenance time vs. supercapacitor charging time curve obtained with three supercapacitors in series one after the other configuration; (**C**) LED turn-on maintenance time vs. supercapacitor charging time curve with three supercapacitors in series in overlapped configuration.

Due to the presence of Ohmic drops within the assembled circuit, the desired potential for the LED was achieved by connecting in series four 100 Ohm resistors with the three Hy_2_SC assemblies. In Figure 6A,B, the equivalent circuit and the three supercapacitors connected in series consecutively were reported.

The curve obtained from the plot of LED turn-on maintenance times in function of supercapacitor charging times showed a linear trend up to 80 s, and the curve achieved a constant value due to the maximum storage capacitance reached.

In a different configuration obtained by overlapping three supercapacitors in series (Figure 6D,E), a linear trend persisted, but with a higher maximum storage capacitance achieved after 150 s.

The last configuration allowed fewer resistors and can be considered a valid option in view of developing wearable devices.

**Figure 7:**
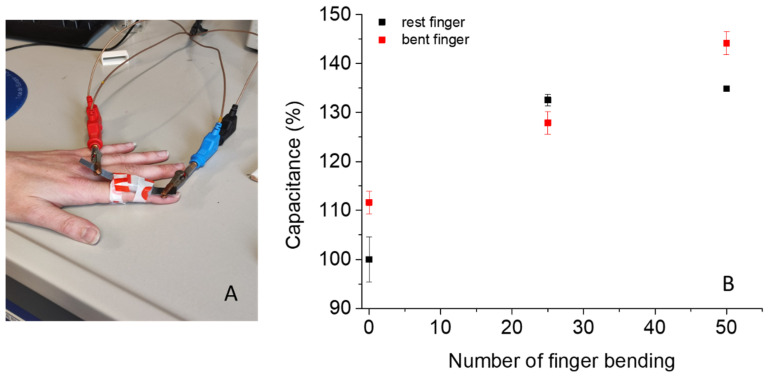
(**A**) Digital image of flexible Hy_2_SC attached to a resting finger and subjected to bending; (**B**) variation of the percentage capacitance of Hy_2_SC before and after 25 and 50 repetitive and consecutive bending stimuli on a finger. Data were obtained in triplicate from three different samples.

To further investigate Hy_2_SC performances under mechanical deformation in a relevant environment, the SC was encapsulated in a silicone shell and subsequentially evaluated in terms of capacitance while undergoing finger bending deformation and after 25 and 50 bending cycles. The charge–discharge curves were recorded with a resting finger (indicated 0°), as shown in Figure 7A, and with the finger bent at 90° to compare Hy_2_SC electrochemical performances while intact or bent and stretched. The considered curves were obtained between 0 and 0.8 V, with five charge–discharge cycles and 25 µA cm^−2^ of current density, with the capacitance values ensuing.

Figure 7B shows the capacitance values expressed as percentages concerning the pristine device measured at 0° after 0, 25, and 50 deformation cycles. These results highlight that the as-prepared supercapacitor exhibited a higher capacitance value when it was bent.

Moreover, capacitance values increase over time and after bending deformations for both bent and relaxed measurements. The supercapacitor not only maintains its performance, but improves it after mechanical deformation. These unexpected behaviors can be ascribed to the boosted electrical contact between the hydrogel interlayer and Grafoil collectors because the charge transfer between electrode and gel plays a key role, as demonstrated by the low performance of the metal collector in the Swagelok cell. On one hand, the compression due to the device bending improves the contact between the electrodes and PANI gel and, at the same time, reduces the charge path in the gel layer. Consequently, the performance is boosted when the device is bent.

On the other hand, mechanical stress could exfoliate the flaky and irregular structure of Grafoil, with the effect of improving the electrical contact between the carbonaceous material and PANI. This phenomenon could lead to deep contact between the two materials and improve performance.

## 3. Conclusions

In this work, appropriately modified PVA-based physical hydrogels have been used to prepare symmetric supercapacitor cells thanks to the self-healing capabilities of PVA. The cheap and reproducible preparation method guarantees strict contact between the interfaces of electrode and electrolyte layers with the consequent minimization of the charge flow resistances. PANI_PAMPSA added to the PVA hydrogel emerges as a good conductive polymer for the preparation of the electronic layers. Comparing the obtained data from the mechanical–morphological and electrochemical characterizations of both semi-cells and symmetric cells that were investigated, 89,000–98,000 MW PVA results as the most promising material for flexible supercapacitor preparation.

The use of flexible Grafoil sheets as charge collectors as an alternative to the Swagelok cell electrodes gives the possibility of achieving values of specific capacities of hundreds of Fg^−1^, comparable with most systems reported in the literature.

Finally, the actual functioning of Hy_x_SC as a supercapacitor has been verified through an assembled circuit in which the switching and keeping on of a red light-emitting diode confirms the ability of this hydrogel system to store and release charge.

Moreover, the stretchability of this kind of material can be an important aspect to further improve the increase in versatility of these systems for biomedical sensors or electrostimulation applications. Therefore, charge–discharge measurements were conducted to obtain capacitance responses while the sample was realistically mechanically deformed by bending and stretching, with an interesting linear capacitance output.

## 4. Materials and Methods

### 4.1. Materials and Characterizations

Commercial poly(vinyl alcohol) (PVA) with different molecular weights, (i) 31,000–50,000 (98.0–98.8% hydrolysis degree), (ii) 89,000–98,000 (+99% hydrolysis degree), were purchased from Merck KGaA, Darmstadt, Germany. Sulfuric acid (H_2_SO_4_, 95.0–98.0%), ammonium persulfate [APS, (NH_4_)_2_S_2_O_8_), ≥98%], and aniline (≥99%) were purchased from Sigma-Aldrich (now Merck KGaA, Darmstadt, Germany); H_2_SO_4_ solution 1.0 M was prepared from dilution of a 95–98% H_2_SO_4,_ Sigma-Aldrich. Aniline was further purified by distillation under nitrogen before use. Poly(2-acrylamido-2-methyl-1-propanesulphonic acid) (PAMPSA), MW ~ 800,000, 10 wt% aq. sol. was purchased from Acros Organics.

A Perkin Elmer Spectrum Two spectrophotometer (resolution of 0.5 cm^−1^ in the range 4000–400 cm^−1^ using 40 scans) was adopted to record the ATR-FTIR spectra. SEM images were taken by a Renishaw field-emission scanning electron microscope, equipped with an InLens detector, operating at 10 kV and a current of 80 pA. Before ATR-FTIR and SEM analysis, the hydrogel samples were washed many times with distilled water, and then placed into a LabConco lyophilizer working at −50 °C and 0.850 mbar for 24 h.

Thermogravimetric Analysis (TGA) was carried out under an inert atmosphere (N_2_) heating from 25 °C to 600 °C, with a scan rate of 20 °C/min with a NETZSCH TG 209F1 Libra instrument. Before the analysis, the samples were kept in the oven for 48 h at 50 °C.

Tensile strength–strain curves were obtained from 40 mm × 10 mm strips by an INSTRON testing machine 5900R with a 1 N load cell at room temperature and 1 mm min^−1^ crosshead speed. Three independent samples were used for each set of hydrogels. The stress *σ* and the strain *ε* were calculated as reported in literature [33] and described in the Supplementary Materials.

Rheological properties of PVA-H_2_SO_4_ hydrogels were determined using an MCR 102 parallel-plate rheometer (Anton Paar, Graz, Austria) as described in our previous work [35]. The water content (Wc%), swelling ratio (Sw%), and porosity of the PVA-H_2_SO_4_ hydrogels were calculated as reported in Supplementary Materials.

### 4.2. Preparation of PVA-H_2_SO_4_ Hydrogel

**Scheme 1:**
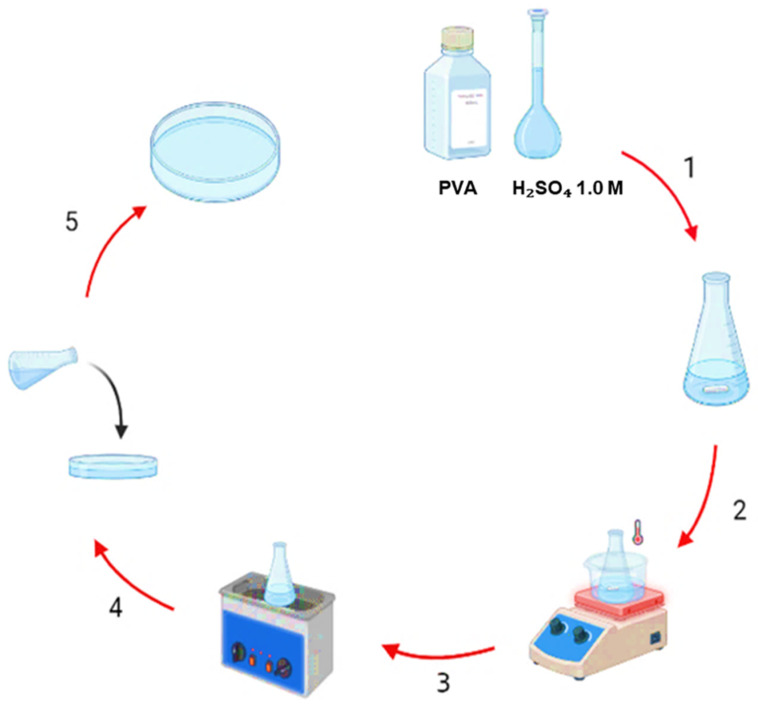
Preparation of Hy_1_ and Hy_2_ layers.

The hydrogel preparation via the freezing–thawing method is described in our previous paper [34] and reported in Scheme 1. Briefly, the PVA was added to 15 mL of aqueous 1.0 M H_2_SO_4_ solution in agreement with the chosen PVA/H_2_SO_4_ weight ratio (optimal PVA/H_2_SO_4_ weight ratio: 1/4 for PVA 31,000–50,000 (Hy_1_) and 1/11 for PVA 89,000–98,000 (Hy_2_)) and heated at 70–90 °C in a water bath under stirring to obtain a transparent solution with reduced presence of bubbles. The solution was then transferred into a Petri dish (8 cm in diameter) and the PVA-H_2_SO_4_ solid hydrogel layer (Hy_x_ where x depends on the molecular weight of PVA used) was obtained after three freeze–thaw cycles between −18 °C for 3 h and room temperature for 1 h

### 4.3. Preparation of PVA-H_2_SO_4_-PANI_PAMPSA Hydrogel, Semi-Cell, and Integrated Hydrogel Supercapacitors

The PVA-H_2_SO_4_-PANI_PAMPSA hydrogel (Hy_x-PANI_PAMPSA,_ green layer), was prepared from a pre-synthetized PANI_PAMPSA polymeric suspension obtained via an oxidative polymerization process [44] and from PVA with different molecular weight solutions (Table 6). Briefly, 1.3 g of PVA was dissolved in 7.6 mL 1.0 M H_2_SO_4_ solution at 80 °C under stirring at 350 rpm (solution A). Then, 1.3 g of PANI_PAMPSA suspension (3.5% *w*/*w* PANI_PAMPSA) was added to solution A after turning off the heating, and kept under vigorous stirring until a homogeneous green solution; then, the mixture was placed in an ultrasonic bath at 70 °C for 6 min.

**Scheme 2:**
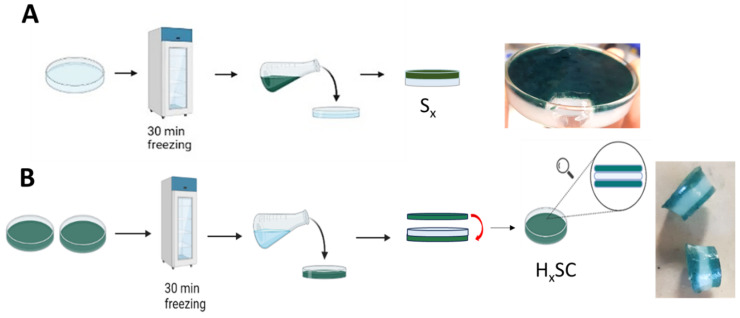
Assembly schemes of (**A**) semi-cell and (**B**) integrated hydrogel supercapacitors.

To obtain the double-layer hydrogel semi-cell (S_x_: Hy_x-PANI_PAMPSA_/Hy_x_), a layer of Hy_x_ (electrolyte) solution was first poured into a Petri dish of 8 cm diameter and subjected to 30 min of freezing. Afterward, a Hy_x-PANI_PAMPSA_ solution layer (electrode) was deposited on the top of Hy_x_ (Scheme 2A). Finally, the double-layer hydrogel configuration was subjected to 6 freeze–thaw cycles. The amount of conductive polymer inside the hydrogel layer is 0.023 g. A symmetric Hy_x_SC was assembled following the procedures previously described. In specific, three hydrogel solutions were prepared in three different flasks, one containing only PVA-H_2_SO_4_ and two also containing the PANI_PAMPSA. The two Petri dishes containing the Hy_x_-PANI_PAMPSA were placed in the freezer at −18 °C for 30 min to ensure a semi-solid consistency. Then, without waiting for its thawing, the electrolyte solution (Hy_x_), kept at room temperature, was poured onto the layer of Hy_x_-PANI_PAMPSA prepared before. Finally, the second layer of Hy_x-PANI_PAMPSA_ was gently overlapped as shown in Scheme 2B. After 6 freeze–thaw cycles, the final compact integrated system (Hy_x_SC) was obtained.

### 4.4. Material Characterizations

A Perkin Elmer Spectrum Two spectrophotometer, (resolution of 0.5 cm^−1^ in the range 4000–400 cm^−1^ using 40 scans) was adopted to record the ATR-FTIR Spectra.

SEM images were taken by a Renishaw field-emission scanning electron microscope, equipped with an InLens detector, operating at 10 kV and a current of 80 pA.

Before infrared spectroscopy (ATR-FTIR) and scanning electron microscopy (SEM), the PVA-H_2_SO_4_ hydrogel samples were first washed many times with distilled water, frozen at –19 °C overnight, and then placed into a LabConco lyophilizer working at −50 °C and 0.850 mbar for 24 h.

Thermogravimetric analysis (TGA) was carried out under an inert atmosphere (N_2_) heating from 25 °C to 600 °C, with a scan rate of 20 °C/min with a NETZSCH TG 209F1 Libra instrument. Before the analysis, the samples were kept in the oven for 48 h at 50 °C.

Tensile strength–strain curves were obtained from 40 mm × 10 mm strips by an INSTRON testing machine 5900R with a 1 N load cell at room temperature and 1 mm min^−1^ crosshead speed. Three independent samples were used for each set of hydrogels.

The stress *σ* and the strain *ε* were calculated as reported in literature [31] and described in the Supplementary Materials.

Rheological properties of PVA-H_2_SO_4_ hydrogels were determined using an MCR 102 parallel-plate rheometer (Anton Paar, Graz, Austria) as described in our previous work [33].

The water content (Wc%), swelling ratio (Sw%), and porosity of the PVA-H_2_SO_4_ hydrogels were calculated as reported in the literature and in detail reported in Supplementary Materials.

### 4.5. Electrochemical Characterization

The ionic conductive properties were obtained from an electrochemical workstation Autolab GSTAT128 N (Metrohm-Autolab) controlled by NOVA 2.10 software via electrochemical impedance spectroscopy (EIS) analysis. A Swagelok-type cell (two 316 stainless steel electrodes model with a testing diameter of 1.0 cm, area = 0.785 cm^2^, Figure S5A) was used. EIS was conducted at room temperature, (25 °C) at the AC voltage amplitude of 10 mV, and frequency range of 0.01–105 Hz. The ionic conductivity (*σ_c_*, *S* cm^−1^) was calculated with the following equation [32]:

(5) $$\sigma_{c}=\frac{s}{RA}$$

where *s* is the thickness of the sample (cm), *R* is the ionic resistance (Ω), and *A* (cm^2^) is the area of the analyzed sample. All the samples investigated were cut with a circular metal mold. A digital caliber was used to measure the sample’s thickness.

The electronic conductive properties were investigated with 4 probe measurements carried out with a Keysight B2902A source meter unit. The inner electrodes measure the voltage while a constant current flow is forced between the two outer electrodes (Figure S5B and detail in Supplementary Materials).

The specific capacitance (*Cp*) was evaluated from galvanostatic charge–discharge (GCD) tests carried out on semi-cells hydrogel at a current density of 0.025 mA cm^−2^ in a voltage range between 0.00 and 0.90 V, according to the equation:

(6) $$Cp=\frac{I\;}{m}\frac{∆t}{∆V}$$

where *I* (A) is the discharge current, *m* (g) is the mass, ∆*t* is the difference between the end-of-discharge time and the end-of-charge time, and ∆*V* is the difference between the end-of-charge potential and the end-of-discharge potential [49].

## Figures and Tables

**Table 1:** Mechanical properties of semi-cells (Sx).

Semi-Cell	Young’s Modulus (MPa)	Strain (%)
S_1_	0.18 ± 0.03	53 ± 4
S_2_	0.05 ± 0.02	170 ± 1

**Table 2:** Wc%, Sw%, and S_PVA_% results.

Sample	Wc%	Sw%	S_PVA_%
Hy_1_	71 ± 2	245 ± 1	10
Hy_2_	77 ± 1	345 ± 9	38
Hy_1_SC	65 ± 2	191 ± 23	8
Hy_2_SC	72 ± 2	264 ± 20	29

**Table 3:** Electrochemical parameters of semi-cells.

Semi-Cell	PANI_PAMPSA Amount (g)	Specific Conductance *κ* (mS/cm)	R (Ohm)	Ionic Conductivity *σ_c_* (S/cm)	Specific Capacitance Cp (mFg^−1^)
S_1_	0.065	13.9 ± 0.4	4.41 ±0.04	0.130 ±0.001	242 ± 3
S_2_	0.023	5.5 ± 0.9	1.25 ± 0.01	0.408 ±0.002	398.0 ± 0.9

**Table 4:** Cp values were obtained with symmetric cells with and without Grafoil.

Symmetric Cell	Applied Current Density (mA)	Cp
Swagelok type cell		
Hy_1_SC	0.025	94 ± 1 (mFg^−1^)
Hy_2_SC	0.025	466 ± 2 (mFg^−1^)
Swagelok type cell with Grafoil		
Hy_1_SC	0.025	13 ± 3 (Fg^−1^)
Hy_2_SC	0.025	73 ± 1 (Fg^−1^)
Hy_2_SC	0.1	65 ± 1 (Fg^−1^)
Hy_2_SC	0.3	48 ± 2 (Fg^−1^)

**Table 5:** Comparison of capacitance value with data reported in the literature.

Symmetric Cell	Capacitance Value	Retain of Specific Capacitance/Number of Cycles	Device	Electrolyte	Ref
Hy_1_SC	26 ± 3 (Fg^−1^)		Flexible SC	1 M H_2_SO_4_	This work
Hy_2_SC	156 ± 38 (Fg^−1^)	78%/500	Flexible SC	1 M H_2_SO_4_	This work
PANI	150–606 (Fg^−1^)	55%/1000	Traditional SC	10^−3^ M HCl ^a^	[57]
PANI + Carbon	144–160 (Fg^−1^)	90%/1000	Traditional SC	1 M H_2_SO_4_ ^a^	[58]
BP/PANI	347–424 (Fg^−1^)	70%/1000	Flexible electrode	1 M H_2_SO_4_ ^a^	[59]
PANI + MWNT	300–430 (Fg^−1^)	68%/1000	Traditional SC	0.5 M H_2_SO_4_ ^a^	[60]
PVA/PANI	571 (Fg^−1^)	75%/2000	Traditional SC	1 M H_2_SO_4_ ^a^	[50]
PVA/PANI	420 (Fg^−1^)	93%/2000	Flexible hydrogel electrode	ATMP	[61]
PVA/TA/PANI/Carbon cloth	102.7 (Fg^−1^)	94.8%/1000	Flexible SC	2.8 M H_3_PO_4_	[62]

^a^ from CCD measures; MWNT = multi-wall carbon nanotube; BP = PANI-coated buckypaper; ATMP = trimethylene phosphonic acid.

**Table 6:** Semi-cell and cell hydrogel composition with PANI_PAMPSA suspension/PVA (*w*/*w*) ratio of 0.5.

Semi-Cell	Integrated Hydrogel Supercapacitor	PVA/H_2_SO_4_ (*w*/*w*)	PVA (%)	PVA MW
S_1_ Hy_1_/Hy_1_-PANI_PAMPSA	Hy_1_SC Hy_1_/Hy_1_-PANI_PAMPSA/Hy_1_	1/4	25	31,000–50,000
S_2_ Hy_2_/Hy_2_-PANI_PAMPSA	Hy_2_SC Hy_2_/Hy_2_-PANI_PAMPSA/Hy_2_	1/11	9	89,000–98,000

## Data Availability

The authors confirm that the data supporting the findings of this study are available within the article and its Supplementary Materials.

## Supplementary Materials

The following supporting information can be downloaded at: https://www.mdpi.com/article/10.3390/gels10070458/s1 (Additional material characterization (ATR; Stress/Strain curves) [32,56,63,64,65]. Figure S1: (A) Swagelok type cell with 316 stainless steel caps of 1.0 cm diameter and configuration for electrochemical measurements; (B) the electronic conductive properties resistance measurements; Figure S2: (A) porosity (%) distribution of WL and GL in Sx; (B) TGA curves of WL and GL of Sx; Figure S3: ATR-FTIR spectra of PVA, Sx-WL Sx-GL and PANI_PAMPSA; Figure S4: Tensile stress/strain curves of S1 and S2; Figure S5: (A) Dynamic viscosity (*η*’) versus angular frequency curve; (B) Frequency sweep curves; (C) Tanδ versus angular frequency curve; (D) Amplitude sweep curves; (E) 3ITT test of PVA-based hydrogels.
