# Supplementary material for: Multi-Layer PVA-PANI Conductive Hydrogel for Symmetrical Supercapacitors: Preparation and Characterization
Source: Gels. 2024 Jul 12;10(7):458. doi: 10.3390/gels10070458 (PMC11276198; doi:10.3390/gels10070458)
Supplement: Supplementary file 1 [file gels-10-00458-s001.zip › gels-3072662-supplementary.pdf]

Supporting Information

S1. Characterizations

Stress and Strain

The stress σ and the strain ε were calculated by the formulas (S1) and (S2) respectively [1]:

$$\sigma = \frac{F}{A} \tag{S1}$$

where *F* (kN) is the force and *A* is the cross-section area (mm^2)

$$\varepsilon = \frac{L - L_0}{L_0} \tag{S2}$$

where L_0 is the initial length and *L* is the final length of the sample in tension.

Water content, swelling ratio, and porosity

To obtain the water content (Wc%) of the different PVA-H_2SO_4 hydrogels, the weight of the hydrogel samples was measured before (*Wi*) and after (*Wd*) drying in an oven at 40 °C for 24 h. The water content was calculated as follows [2,3]:

$$Wc\% = \frac{Wi - Wd}{Wi} 100 \tag{S3}$$

To estimate the degree of swelling, the dried samples were immersed in distilled water at 25 °C for 72 h to reach swelling equilibrium. The samples were withdrawn from the bath, and the excess of unabsorbed water was quickly removed with filter paper before being weighed on an analytical balance. The swelling ratio (Sw%) was calculated according to the following formula [2,3]:

$$Sw\% = \frac{Ws - Wd}{Ws} 100 \tag{S4}$$

where *Ws* is the weight of swollen gel, *Wd* is the weight of dry gel.

Focusing on the PVA amount ($S_{PVA}\%$), the swelling data were normalized on the polymer fraction (f) in the hydrogel according to the following formula [4].

$$S_{PVA}\% = \frac{Sw\%}{f} \tag{S5}$$

The porosity of the dried samples was determined from eq. (S6) by measuring the adsorbed volume of cyclohexane respect to the total volume of each sample using a pycnometer.

$$Porosity\% = \frac{Vpores}{Vsample}\,100 = \frac{mw-md}{m1-m2+mw}100 \qquad \text{(S6)}$$

where *md* is the mass of the dry gel, *mw* is the mass of the wet hydrogel in cyclohexane, *m1* is the mass of the apparatus filled with cyclohexane, and *m2* is the mass of the apparatus with cyclohexane and hydrogel.

Electrical properties

The resistance (R) was calculated with the Ohm's law and the sheet resistance (R■) is equal to:

$$R_{\blacksquare} = R\,\frac{W}{L} \qquad \text{(S7)}$$

W and L are the sample's width and length, respectively.

The specific resistance (ρ) can be calculated by:

$$\rho = R_{\blacksquare}\, t \qquad \text{(S8)}$$

where t is the thickness of the sample.

The specific conductance (κ) is calculated by:

$$k = 1/\rho \qquad \text{(S9)}$$

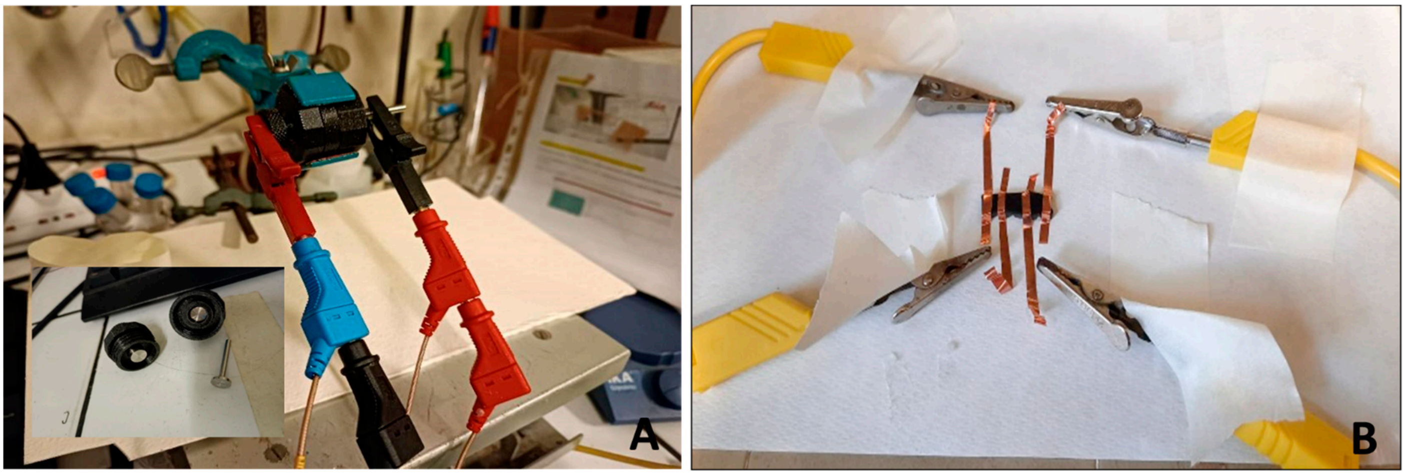

Figure S1. A) Swagelok type cell with 316 stainless steel caps of 1.0 cm diameter and configuration for electrochemical measurements; B) the electronic conductive properties resistance measurements.

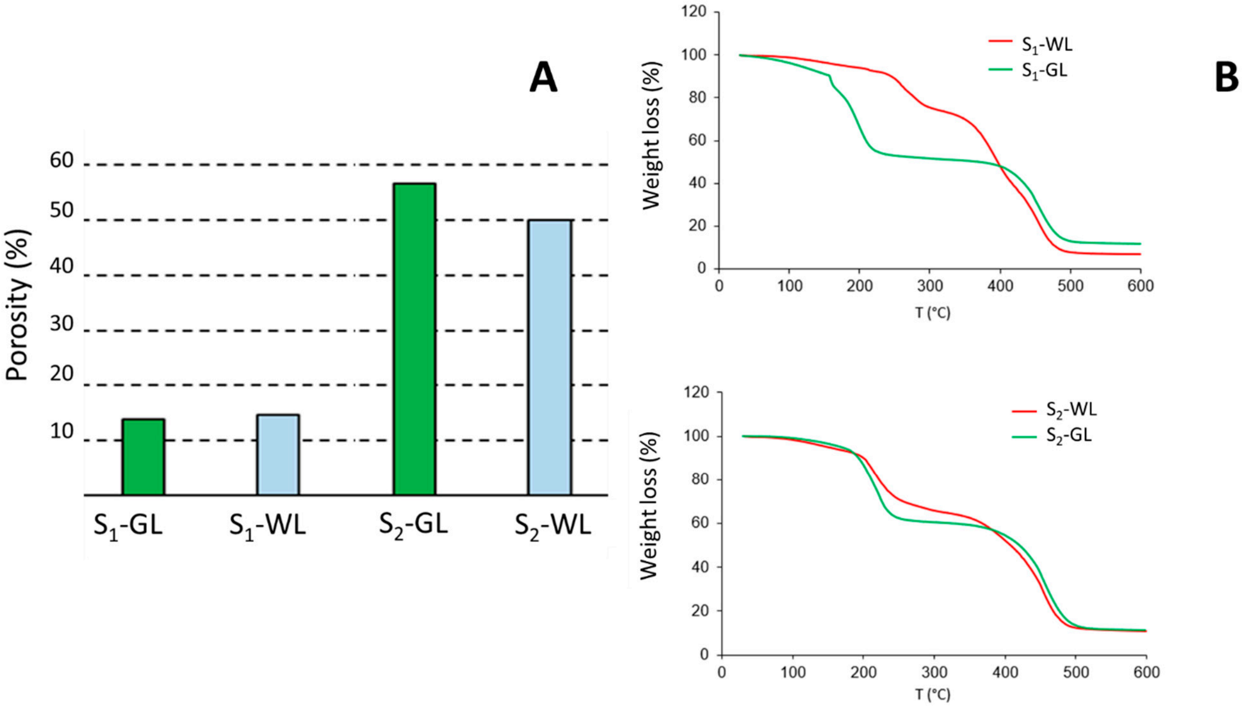

Figure S2. A) porosity (%) distribution of WL and GL in Sx; B) TGA curves of WL and GL of Sx.

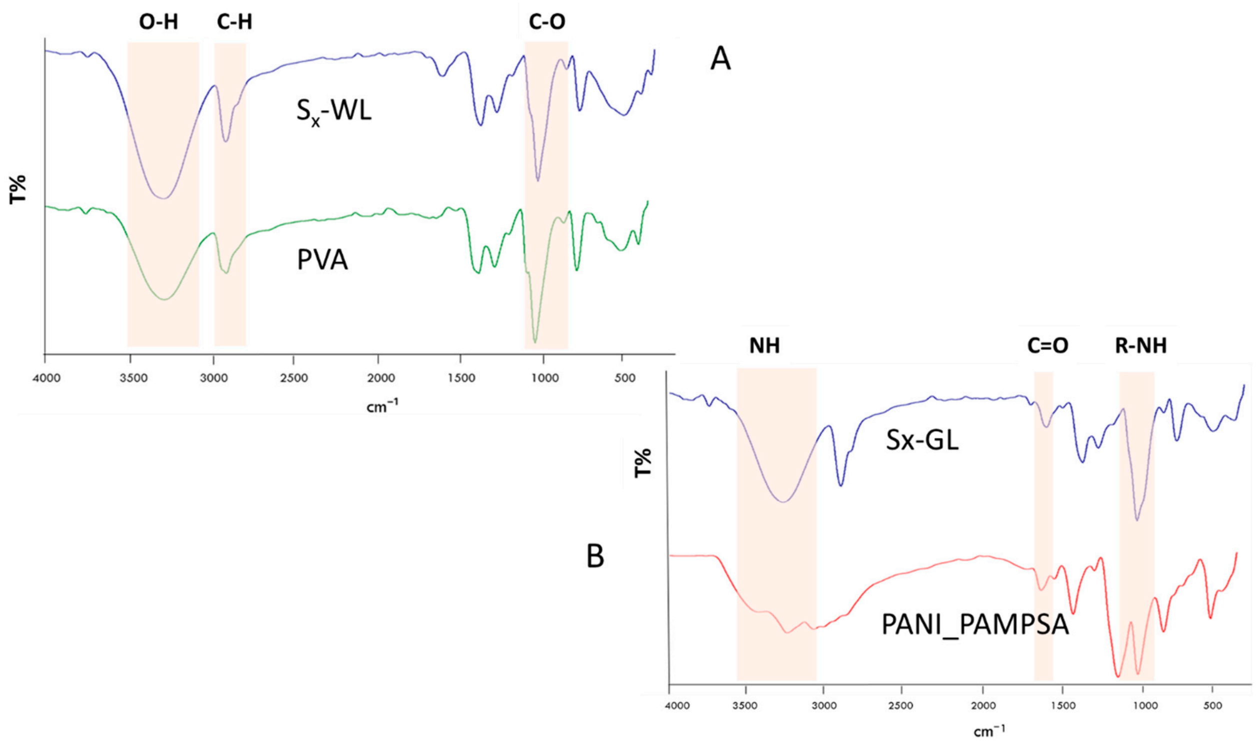

Figure S3. ATR-FTIR spectra of PVA, S_x-WL S_x-GL and PANI_PAMPSA.

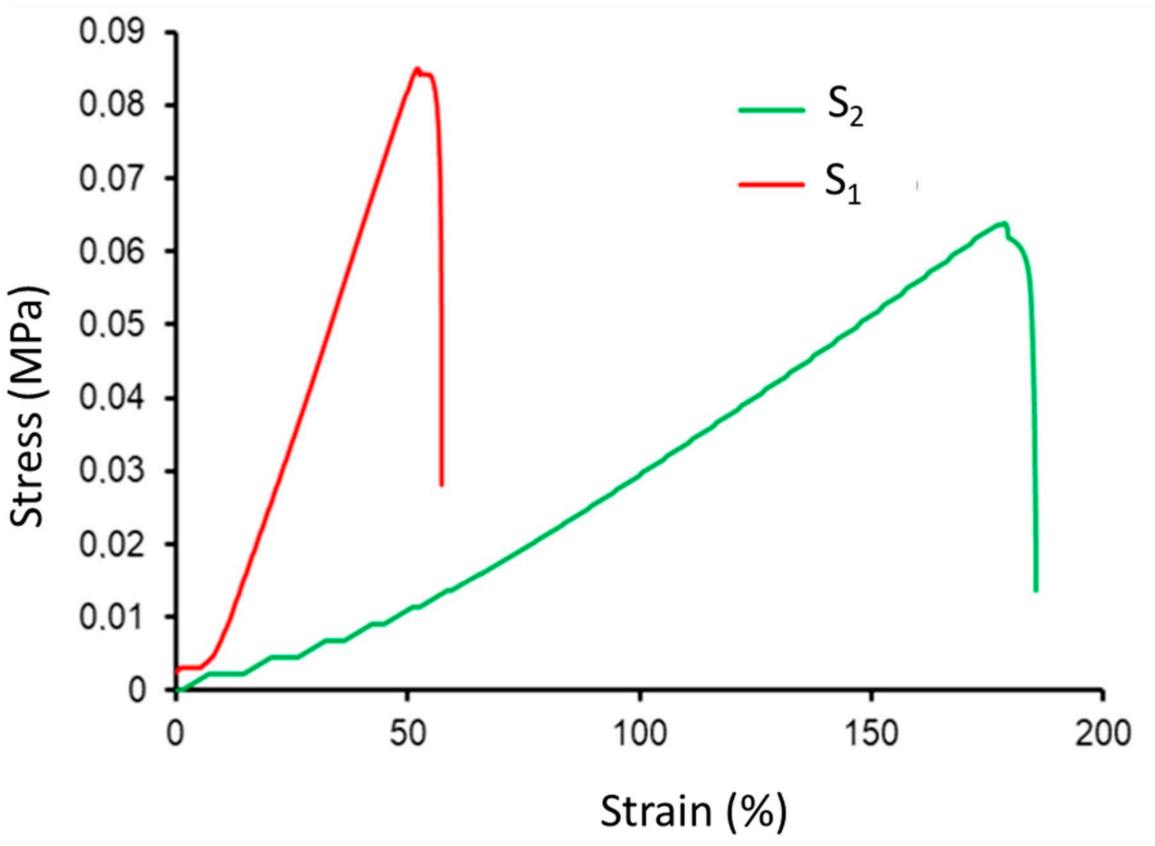

Figure S4. Tensile stress/strain curves of S_1 and S_2.

S2. Self-healing property

Rheological properties of PVA-H_2SO_4 hydrogels were determined using an MCR 102 parallel-plate rheometer (Anton Paar, Graz, Austria) in a plate-plate geometry with a diameter of 25 mm (PP-25 plate) and a gap of 1.2 mm. Disks of the hydrogel with a diameter of 25 mm and a thickness of 1.2 mm were deposited onto the plate of the rheometer. Subsequently, the upper plate was lowered until it contacted the sample's surface. The excess material was removed with a spatula, and the trap was filled with distilled water to avoid evaporation phenomena. An oscillatory frequency sweep test was carried out to assess the self-healing property of hydrogels at 25 °C in a frequency range from 500 to 0.1 rad s^{-1}, with a fixed strain value equal to 1 %. Oscillatory amplitude sweep analysis was performed at 25°C to assess the stress value at which chains flow and the interactions are broken. The frequency value was kept constant at 1 rad s^{-1}, while the amplitude of the shear strain varied from 0.01 to 1000 %.

Finally, a three-interval thixotropic test (3ITT) was carried out in controlled shear rate (CSR) mode to assess the hydrogel's mechanical properties after the application of high shear rates: the rest condition of the hydrogel was simulated by applying a low shear rate (0.1 s^{-1}) at 25 °C; then, a high shear rate (100 s^{-1}) was applied to simulate the breaking of chain interactions, and finally, the resting conditions were restored to evaluate the % recovery of materials property. The value of the high shear rate applied (100 s^{-1}) was chosen on the base of the amplitude sweep test results.

The rheological frequency sweep was conducted at 25°C, and the dynamic viscosity (η') curve (Fig. S5) showed that η' tends to a plateau value at low frequencies. The absolute value of the slope of the initial η', equal to 0.07, confirms the Newtonian character of the hydrogel at low frequency, typical of self-healing materials (pseudoplastic fluids) [5]. Furthermore, frequency sweep tests were conducted, and storage modulus (G′) and loss modulus (G″) were recorded (Fig. S5B). The results suggest that the dynamic bond-based gel network forms a pseudo-structure with minimal steric hindrance for the chain flow, owing to the short lifetime of the dynamic bond. Indeed, a tf value of

0.03 seconds was calculated for Hyx, given by the reciprocal value of the crossover frequency, confirming that the chain flow relaxation strongly contributes to self-healing on a reasonable time scale. No self-healing materials exhibit low chain relaxation times, making it impossible to observe and calculate tf. The trend of the loss factor (tanδ) was reported as a function of the angular frequency. Figure S5C shows an upward trend of tanδ, confirming the hydrogel liquid-like behaviour, because of the chain mobility and/or bond exchange, linked to the self-healing property. Finally, an oscillatory amplitude sweep test was performed to determine the breaking point of the Hyx. The crossover between G′ and G″ occurred with 4.7 % of applied shear strain (corresponding to 8.2 Pa of applied shear stress) (Fig. S5D). This information was utilized to perform the 3ITT analysis (Fig. S5E). From the curve, it was possible to calculate the % recovery of the viscosity of the hydrogel after the high shear rate applied, which amounted to 64% after 2 minutes. The obtained results suggested that Hyx completely fulfilled the three required criteria, from a rheological point of view, to be classified as a self-healing material.

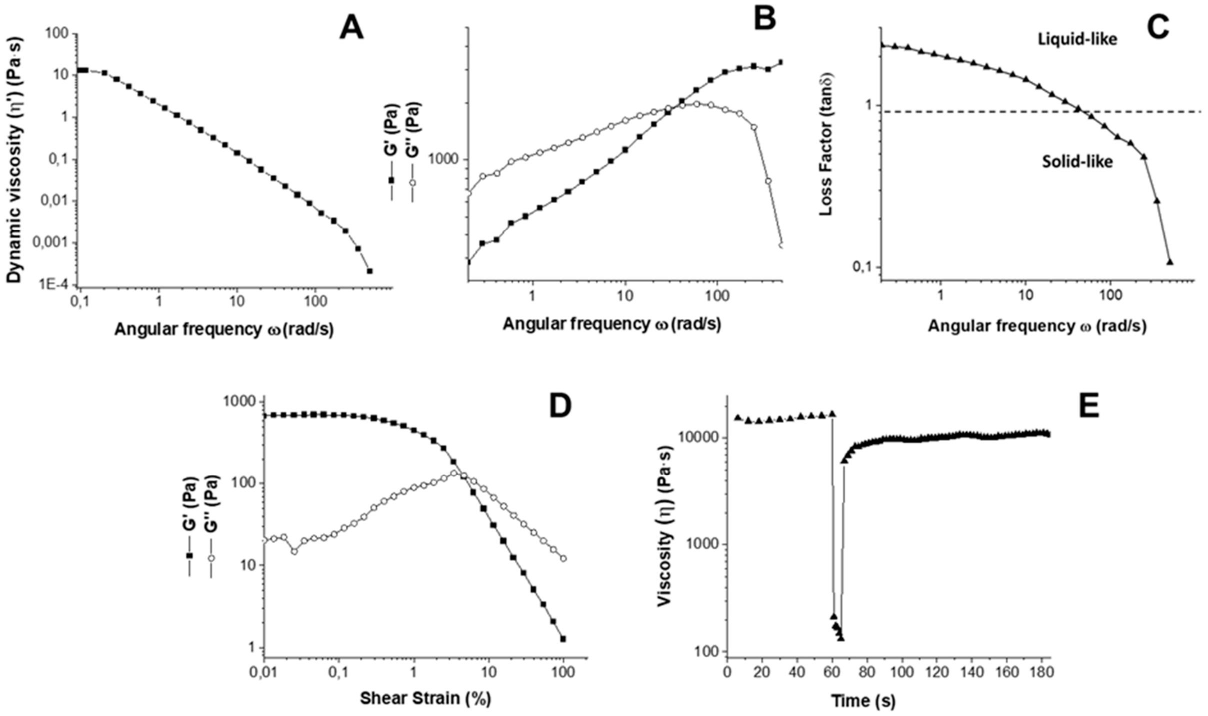

Figure S5. A) Dynamic viscosity (η') versus angular frequency curve; B) Frequency sweep curves; C) Tanδ versus angular frequency curve; D) Amplitude sweep curves; E) 3ITT test of PVA-based hydrogels

References

[1] W. Bin Ma, K.H. Zhu, S.F. Ye, Y. Wang, L. Guo, X.Y. Tao, L.T. Guo, H.L. Fan, Z.S. Liu, Y.B. Zhu, X.Y. Wei, A self-healing hydrogel electrolyte towards all-in-one flexible supercapacitors, Journal of Materials Science: Materials in Electronics 32 (2021) 20445–20460. https://doi.org/10.1007/s10854-021-06555-5.

[2] L. Zhang, Z. Wang, C. Xu, Y. Li, J. Gao, W. Wang, Y. Liu, High strength graphene oxide/polyvinyl alcohol composite hydrogels, J Mater Chem 21 (2011) 10399–10406. https://doi.org/10.1039/c0jm04043f.

[3] S. Tanpichai, K. Oksman, Cross-linked nanocomposite hydrogels based on cellulose nanocrystals and PVA: Mechanical properties and creep recovery, Compos Part A Appl Sci Manuf 88 (2016) 226–233. https://doi.org/10.1016/j.compositesa.2016.06.002.

[4] T. Mecca, M. Ussia, D. Caretti, F. Cunsolo, S. Dattilo, S. Scurti, V. Privitera, S.C. Carroccio, N-methyl-D-glucamine based cryogels as reusable sponges to enhance heavy metals removal from water, Chemical Engineering Journal 399 (2020) 125753. https://doi.org/10.1016/j.cej.2020.125753.

[5] M. Shin, S.H. Shin, M. Lee, H.J. Kim, J.H. Jeong, Y.H. Choi, D.X. Oh, J. Park, H. Jeon, Y. Eom, Rheological criteria for distinguishing self-healing and non-self-healing hydrogels, Polymer (Guildf) 229 (2021) 123969. https://doi.org/10.1016/j.polymer.2021.123969.
